# Forearm Compartment Syndrome following Thrombolytic Therapy for Massive Pulmonary Embolism: A Case Report and Review of Literature

**DOI:** 10.1155/2011/678525

**Published:** 2012-01-23

**Authors:** Ravi Badge, Mukesh Hemmady

**Affiliations:** Department of Trauma and Orthopaedics Surgery, Wrightington, Wigan and Leigh NHS Trust, Wigan WN1 2NN, UK

## Abstract

Use of thrombolytic therapy in pulmonary embolism is restricted in cases of massive embolism. It achieves faster lysis of the thrombus than the conventional heparin therapy thus reducing the morbidity and mortality associated with PE. The compartment syndrome is a well-documented, potentially lethal complication of thrombolytic therapy and known to occur in the limbs involved for vascular lines or venepunctures. The compartment syndrome in a conscious and well-oriented patient is mainly diagnosed on clinical ground with its classical signs and symptoms like disproportionate pain, tense swollen limb and pain on passive stretch. However these findings may not be appropriately assessed in an unconscious patient and therefore the clinicians should have high index of suspicion in a patient with an acutely swollen tense limb. In such scenarios a prompt orthopaedic opinion should be considered. In this report, we present a case of acute compartment syndrome of the right forearm in a 78 years old male patient following repeated attempts to secure an arterial line for initiating the thrombolytic therapy for the management of massive pulmonary embolism. The patient underwent urgent surgical decompression of the forearm compartments and thus managed to save his limb.

## 1. Background

Acute massive pulmonary embolism (PE) is an uncommon clinical entity but carries an exceptionally high mortality. A rapid diagnosis of massive PE is very crucial to initiate the potentially life-saving therapy. The use of thrombolysis in conjunction with standard anticoagulation in the acute phase has been shown to reduce the mortality rate in this group of patient [1]. According to International Cooperative Pulmonary Embolism Registry (ICOPER), there is no significant difference in the mortality and recurrence of PE in patients treated with thrombolytic therapy compared with standard intravenous heparin infusion. However, these findings were not applicable for patients with right ventricular dysfunction and unstable haemodynamic condition [2, 3].

Due to its fatal haemorrhagic complications, the thrombolytic therapy has been strictly recommended for the patients with proven massive PE. The rate of significant bleeding has been reported to be around 22–45%. The bleeding most commonly occurs at the vascular catheter site, viscera, and intracranium. Although management for minor bleeding has been supportive, serious bleeding does warrant withdrawal of the thrombolytic therapy. Isolated cases of compartment syndrome after thrombolytic therapy have been reported in the literature [4]. It is more commonly associated with either minor trauma or in the limb used for an intravenous access. We report the case of an isolated right forearm compartment syndrome in an unconscious patient who received the thrombolytic therapy for an acute massive pulmonary embolism.

## 2. Case Report

A 78-year-old male presented to the hospital with the history of fever and breathlessness. After initial assessment and investigations, the diagnosis of chest infection was made, and the patient was admitted to short stay unit for further management. The patient was a known case of myasthenia gravis and had history of asbestosis in the past. Whilst being on ward, the patient suddenly collapsed and required resuscitation. The anaesthetist was called to intubate the patient and was then transferred to the Intensive care Unit (ICU) for further management. After initial stabilisation, the patient was investigated to find the cause of sudden collapse. The ECG showed the right bundle branch block. The patient had raised Troponin T level, and the echocardiogram revealed well-preserved left ventricular function with reduced right ventricular function and bright mass in pulmonary artery, thus confirming the diagnosis of massive pulmonary embolism.

**Figure 1:**
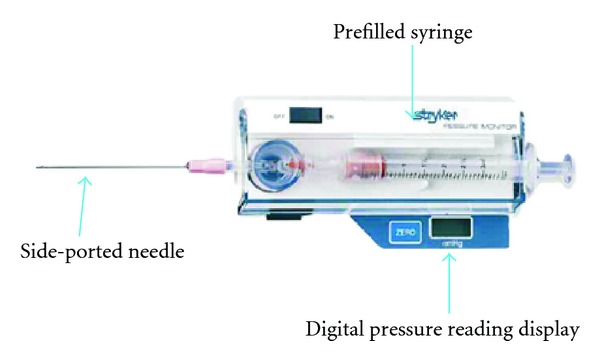
Stryker Intracompartment pressure-monitoring device.

The patient was thrombolysed using 100 mg of tissue plasminogen activator (tPA) over two hrs and Heparin 1000 units per mL at 1 mL/hr given in separate lines. The nor-adrenaline was used to maintain mean arterial pressure of around 85. The patient had femoral arterial line after numerous failed attempts to have a right radial artery line. The patient responded very well to the thrombolytic treatment leading to stable haemodynamic condition. Eight hours following the initiation of thrombolytic therapy, the right forearm of the patient was noted to be very swollen and tight. The orthopaedic team was called immediately to assess the forearm in view of compartment syndrome. As patient being intubated, it was difficult to diagnose a compartment syndrome on just clinical ground, and hence a universally accepted, calibrated handheld device (Stryker, Kalamzoo, Michigan) was used to measure the compartment pressure in the involved forearm compartments (Figure 1). The pressure was measured to be 45 mmHg. The decision of an emergency fasciotomy of the involved forearm was taken following this measurement and high index of suspicion.

**Figure 2:**
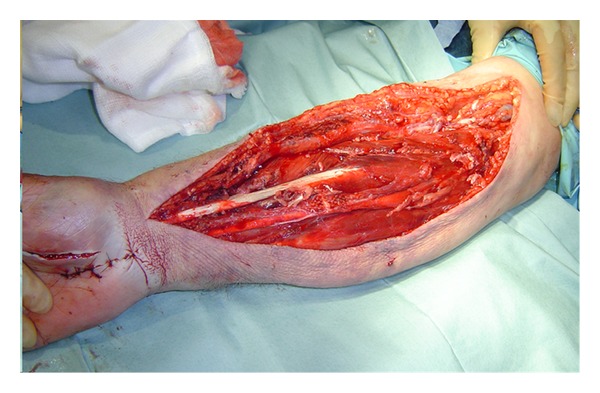
Fasciotomy with extensile Henry's approach for forearm compartment syndrome.

**Figure 3:**
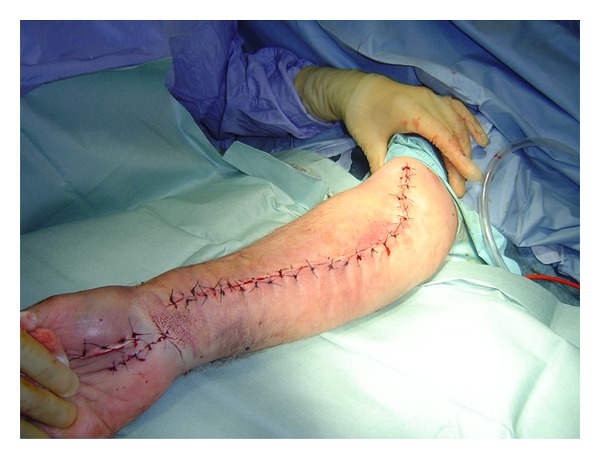
Delayed primary closure of the fasciotomy wound.

 The fasciotomy of the forearm was performed by extensile Henry's approach along with decompression of carpel tunnel and abductor compartment of the hand (Figure 2). The muscles within both superficial and deep compartment were bulging and bloodstained. During this procedure, the massive blood clot was found in the volar compartment of the forearm, close to the radial artery puncture mark and was carefully evacuated. Flexor digitorum profundus was found to be partially necrotic and was therefore debrided till bleeding muscle identified. The wounds of carpel tunnel and adductor compartment release were closed primarily, and volar forearm wound was left open. After 72 hrs, the fasciotomy wound was then evaluated again and closed satisfactorily without any tension on the suture lines (Figure 3). The patient made remarkable recovery postoperatively and was then commenced on vigorous physiotherapy.

 At the end of 3-month followup, the patient had full range of movements in elbow, terminal restriction of movement in wrist and hand. He had functional muscle power in his intrinsic muscles of the hand. Apart from mild tingling in the hand, there was no sensory deficit.

## 3. Discussion

Acute compartment syndrome occurs as a result increased interstitial pressure in closed osseous fascial compartment thus compromising the circulation and function of the tissues within that compartment. The initial insult either in the form of trauma, internal bleeding, or ischaemia leads to swelling within the closed compartment and compromising the tissue perfusion. This sequence of event causing tissue ischaemia if not reversed in time by decompression of compartment can lead to necrosis of the tissue [5]. In our case, the repeated attempts made to secure an arterial line in order to administer the thrombolytic therapy led to an extravasation of blood into forearm compartments and thus leading to an increased compartment pressure.

The diagnosis of compartment syndrome is usually made on clinical suspicion when patient complains of a disproportionate pain in the involved limb and elicits the severe pain on passive stretch of the muscle within the involved compartment. It is very difficult to make this judgement in a patient with a head injury, unconscious or on artificial ventilation like our case. In such scenario, measurement of intracompartmental pressure with the calibrated device along with clinical suspicion helps to make the diagnosis of compartment syndrome [6]. The Stryker intracompartmental pressure-monitoring device is a convenient, self-contained, and reliable unit for an instant or continuous measurement of compartment pressure and has been used worldwide for measuring compartment pressure. Boody and Wongworawat [7] demonstrated acceptable levels of accuracy and precision of Stryker hand held device as an objective method for the measurement of intracompartmental pressure, which can be used in the diagnosis of compartment syndrome. 

McCarthy et al. [8] in their combined radiological and cadaveric study have revealed the safest approach to measure the pressure in the volar compartment of the forearm. According to this study, an approach from the midline to the ulna, between the tendons of the Flexor carpi radialis and Palmaris longus considered to be the safest. This method allows the placement of catheter in the deep volar compartment of the forearm without any inadvertent damage to the neurovascular structures. In our case, it was difficult to assess the clinical signs of compartment syndrome as patient was artificially ventilated. Therefore, it is recommended to have strong clinical suspicion and objective assessment of compartment pressure in the similar situations [9]. It is important to act promptly and decompress the involved compartment by doing the adequate fasciotomy once the diagnosis is made. Delay in diagnosis and decompression of compartment syndrome more than eight hours can lead to irreversible damage to the skeletal muscles and nerves in the involved compartment [5].

It has been well documented in literature about the complications of bleeding with thrombolytic therapy administered to patients with myocardial infarction and stroke but not much data about the patients with massive pulmonary embolism. The complication rate for bleeding due to thrombolytic therapy seems to be higher in elderly population [10–12]. In massive pulmonary embolism, lysis of thrombi can be achieved quicker with the appropriate thrombolytic therapy than with conventional heparin therapy in a patient with unstable haemodynamic condition and reduced right ventricular function [1–3]. But it needs to be administered with great caution in elderly patients due to increased risk of bleeding especially at the site of venepuncture and arterial lines [11].

McQueen et al. [13] emphasized the importance of prompt diagnosis and treatment after acute compartment syndrome and also stressed that awareness of this clinical entity among the nursing and medical staff is the most important factor contributing to an early diagnosis. Thus, heighten awareness about the acute compartment syndrome in an intubated patient following thrombolytic therapy can avoid long-term disability related with compartment syndrome. Therefore, such high-risk patients should be thoroughly evaluated using pressure-measuring device, and urgent fasciotomy of the involved compartments should be performed. In our case, the success of the treatment lies in an appropriate referral from the ICU doctors, an urgent diagnosis, and emergency fasciotomy undertaken by an orthopaedic team.

## 4. Conclusion

It is imperative for medical staff working on ICU setup to be aware of the possibility of compartment syndrome in a patient on thrombolytic therapy. One has to be very careful in taking vascular access in such group of patient to avoid extravasation of blood leading to increased intracompartmental pressure. Prompt referral should be made to the orthopaedic team to assess the tense swollen limb in a patient treated with thrombolysis for massive pulmonary embolism.
